# Development and first application of an audit system for screening programs based on the PRECEDE-PROCEED model: an experience with breast cancer screening in the region of Lombardy (Italy)

**DOI:** 10.1186/s12889-020-09842-8

**Published:** 2020-11-25

**Authors:** Danilo Cereda, Antonio Federici, Angela Guarino, Grazia Serantoni, Gruppo PRECEDE-PROCEED, J. A. Bastiampillai, E. Gabrielli, F. Grimaccia, L. Tessandri, M. Schivardi, M. Crisetig, R. Bardelli, G. Gola, E. Anghinoni, A. Bozzeda, S. Gotti, L. Cavalieri D’Oro, A. Ilardo, G. Moretti, F. Lobuono, E. Merlo, A. Silvestri, G. Beghi, R. Lucchini, G. Marazza, E. Rossetti, N. Leonardo, E. Tidone, A. Lamberti, P. Ceresa, L. Acerbi, L. Camana, G. Magenes, L. Cecconami, A. M. Cioccarelli, Liliana Coppola, Patrizia Lemma, Paolo Giorgi Rossi

**Affiliations:** 1 DG Welfare, Regione Lombardia, Milano, Italy; 2 Direzione Generale della Prevenzione, Ministero della Salute, Rome, Italy; 3 Dipartimento di Psicologia, Università di Roma “La Sapienza”, Rome, Italy; 4 ASL Roma 5, Tivoli, Italy; 5 Gruppo PRECEDE-PROCEED, Regione Lombardia, Milano, Italy; 6 Università degli Studi di Torino, Torino, Italy; 7 Azienda Unità Sanitaria Locale – IRCCS di Reggio Emilia, Reggio Emilia, Italy

**Keywords:** Breast cancer, Mass screening, Health intervention planning, PRECEDE-PROCEED model, audit

## Abstract

**Background:**

High participation and performance are necessary conditions for the effectiveness of breast cancer screening programs. Here we describe the process to define and test a planning software application and an audit cycle based on the PRECEDE-PROCEED model applied to improving breast cancer screening.

We developed a planning software application following the phases of the PRECEDE-PROCEED model. The application was co-designed by local cancer screening program coordinators. An audit model was also developed. The revised application and the audit model were tested by all the coordinators of 15 breast cancer screening programs in the region of Lombardy in a 3-day workshop. The project plans produced using the application were compared with those produced in the previous year for clarity and completeness.

**Results:**

The 9 phases of the PRECEDE-PROCEED model were adapted to screening as follows: 1) identification of program goals (i.e., participation, sensitivity, false positive); 2) epidemiological issues; 3) best practices analysis; 4) evidence-based actions to be implemented in the screening center and the relationships with partners and stakeholders; 5) priority setting and identification of solutions for each issue; 6) definition of indicators; 7) monitoring; 8) evaluation; 9) impact assessment. The application automatically generated reports for each phase. During the audit cycle, the regional health authority negotiated the targets to be reached with local authorities and collected the improvement plans generated by the application. The plans produced after the application was adopted were more standardized and had clearer indicators for monitoring and evaluation compared to those produced in the previous year.

**Conclusions:**

The software application helps standardize criteria for planning interventions to improve screening programs and facilitates the implementation of the audit cycle.

**Supplementary Information:**

The online version contains supplementary material available at 10.1186/s12889-020-09842-8.

## Background

In Italy breast cancer screening has been recommended since 1999. The Regions have received a mandate to implement programs to actively invite women aged 50–69 to perform a mammography every 2 years [1]. Nevertheless, the activation of screening programs has been slow; in 2015, on average 18% of the target population was not regularly invited for a screening test, with strong differences between geographical areas (ranging from 3% in the North to 40% in the South). Furthermore, participation has been low, ranging from 31 to 77% among the regions [2].

Many studies, both international [3, 4] and Italian [5–8], have shown that organized screening has a more appropriate diagnostic pathway and better performance than does spontaneous or opportunistic screening. Furthermore, at least in European countries, organized programs showed to obtain higher test uptake [4] thus a stronger impact on breast cancer mortality in the target population. Even if the best way to measure the effectiveness of the screening program in informing the target population should be monitoring informed choice of the women, it is difficult to measure it, thus the European Council Initiative on Breast Cancer supported the use of participation as a good proxy of informed choice [9, 10] for the age group where the guidelines made a strong recommendation in favor of screening [11].

Previous systematic reviews identified several determinants and interventions which proved to be effective in increasing the uptake of mammography, including mail reminders, telephone calls, GP signature on the invitation letter, pre-fixed appointment compared to open invitation to fix an appointment, and strategies to reduce logistical barriers [12, 13].

In 2014, order to promote effective interventions to increase participation in organized cancer screening programs and to improve their implementation and performance, the Italian Ministry of Health started a project (“Implementation of screening programs: analysis of barriers and facilitating factors, modifiable and not” [14]) coordinated by AGENAS (the National Agency for Regional Health Services) and in collaboration with a group of regions that agreed to participate with the aim of understanding and overcoming the barriers to screening implementation.

According to the WHO indications [15], the project was inspired to the stewardship principles. Stewardship refers to the wide range of functions carried out by governments as they seek to achieve national health policy objectives [16]. It aims at: generating intelligence; formulating strategic policy direction; ensuring tools for implementation such as powers, incentives, and sanctions; building coalitions and partnerships; ensuring a fit between policy objectives and organizational structure and culture; ensuring accountability.

One of the aim of the stewardship action was to provide the regions and the local screening programs with tools that could help planning of improvement actions. The logical framework adopted for these tools was the PRECEDE-PROCEED model [17] an operational approach developed to define and implement complex health promotion interventions.

The PRECEDE-PROCEED model is widely used to support the process of planning and evaluating actions in the field of health promotion with a multi-dimensional and multi-disciplinary structure. The PRECEDE-PROCEED model assumes that health and health behaviors are influenced by multiple factors - epidemiological, socio-psychological, administrative, political, environmental – which must be considered and assessed for their modifiability in order to ensure effective interventions.

### PRECEDE PROCEED AUDIT: the national experience

The aims of the project were to:

1. analyse barriers and factors which facilitate the implementation and participation in cancer screening programs in three Italian macro-areas (North, Center, South);
2. provide support for screening planning in order to overcome regional and local differences in the implementation of the programs;
3. develop a tool for planning screening programs based on the PRECEDE-PROCEED model, favouring the implementation of interventions based on the best available evidence and tailored to the specific local context.

In the first part of the project, AGENAS used some available data sources (National Health Interview [18] behavioral risk factor national survey [19], and official statistics from the Centre for Screening Monitoring [2]) to conduct an analysis of screening test uptake and screening program implementation in Italy. The analysis showed strong differences between northern and southern Italy, but it also identified modifiable barriers and facilitating factors, such as the presence of opportunistic screening.

During the project, AGENAS contacted all the regional health authorities for a survey on regional screening organization, management of screening, and stewardship toward the local health authorities for cancer screening programs. The results highlighted the regional governments’ poor support of local screening programs.

The project had two outputs: 1) an executive summary containing a description of barriers and facilitating factors; 2) a tool, based on the PRECEED-PROCEDE model, which helps planning actions to improve screening programs [20].

The tool developed at the end of the AGENAS project was a planning application that was tested in 7 Italian Local Health Authorities (LHAs), including 3 LHAs in region of Lombardy [20]. The test included a preparatory phase in which the LHAs were asked to collect data and documents, a site visit, and then a phase providing the possibility of using the application for planning actions to improve the screening program.

### PRECEDE-PROCEED AUDIT: the region of Lombardy experience

The General directorate of Welfare of Region of Lombardy (DGW-LR), starting from the AGENAS pilot project, decided to launch a program to improve breast cancer screening implementation and participation by designing a peer-to-peer audit system and a software application to help planning interventions to improve screening programs at local level.

The aim of this study is to describe the experience of the region of Lombardy Health System in defining and testing usability a software application and an audit cycle based on the PRECEDE-PROCEED model.

## Implementation

### Setting

According to the Italian guidelines [1], the organization of breast cancer screening programs is mandatory for all regional health systems in Italy. The Italian guidelines are based on the “Quality Assurance European Guidelines” [21] according to which: the target population is all women aged 50–69 and the screening test is a mammography in two projections, with double reading, performed every 2 years.

The Region of Lombardy is located in Northern Italy, the wealthiest part of the country, and is the most populated region in Italy (roughly 10 million inhabitants). Its regional health system, even if inspired by the same equity and universalistic principles, is quite different from those of other Italian regions and it is characterized by a strict separation of the provider function, performed by public or private hospitals, from the third payer and control function, administered by the Local Health Authorities (LHA). The DGW-LR has the duty to guarantee oncologic screening programs targeting the whole population of region of Lombardy. The DGW–LR coordinates and supports the 8 LHAs which include 15 screening centers. The LHAs organize the screening programs, but the diagnostic testing and treatment (mammograms, biopsies and surgical procedures) are performed in accredited private and public hospitals. The two entities are therefore administratively independent (Table 1).

**Table 1:** The public health and screening organization in Region of Lombardy

		Functions
National Healthcare System	Italian Minister of Health (IMH)	The parliament sets the Essential Levels of Care (LEA) and the National Health Fund. The IMH, with the support of the National Screening Monitoring Centre, agrees with regional governments the operational definition of the screening LEA, the monitoring of LEA-related indicators, objectives for implementation and quality assurance, and budget criteria for fund allocation (linked to objectives). The National Plan of Prevention, the national guidelines for screening and the screening reporting system are the main tools for this task.
Healthcare System of the Region of Lombardy	General directorate of Welfare of Region of Lombardy (DGW-LR) Prevention Unit	-defines budgets, rules, objectives for Local Health Authorities, which are the payers, and for hospital and clinic trusts (called Territorial Health Social Trusts), which are the providers; - coordinates and supports Local Health Authorities; - manages regional “monitoring system” (database) to guarantee oncologic screening to all citizens of the Region of Lombardy evaluating data reported by the LHAs; -reports performance indicators to IMH.
	8 Local Health Authorities (LHA) – including 15screening centers	- make agreements with the Territorial Health Social Trusts and with the hospitals with regard to the number of medical exams of screenings (mammograms, fecal immunologic tests, colonoscopies, pap tests, etc) - control the quality of medical exams -report data to DGW-LR - manage the “call-recall system” (send letters, recall, manage website, etc.) -manage communications with patients (by e-mails, call center) -elaborate performance and early outcome population-based indicators - evaluate the performance of each hospital and refer the data of activities to DGW-LR.
	public accredited hospitals (27) and private accredited hospitals	- do medical exams of screening, assessment and treatment - report data to LHAs

### DEVELOPMENT AND TEST of the audit program and software application

The DGW-LR identified a coordinator of the audit program and a working group that included screening program coordinators and some external experts who had previously collaborated on the AGENAS project [14]. The working group developed a new audit model and a new application.

To develop a model specific to the cancer screening setting, the following three sources were reviewed: the audit model used in Region of Lombardy to assess food safety (every year the DGW-LR audits the LHAs) [22]; a short list of references identified through a scoping review of the scientific literature [13, 23–28]; the results of the AGENAS pilot project.

A new planning application based on the PRECEDE-PROCEED model was developed starting from the AGENAS pilot project. All phases of the PRECED-PROCEED model were reanalyzed and readapted by DGW-LR to the screening setting of the Region of Lombardy. Furthermore, a Microsoft Access macro was programmed to render the PRECEDE-PROCEED model more user-friendly and to automate the compilation of reports.

The audit model and the new application were revised with the help of all the coordinators of the 15 screening centers in the region of Lombardy over the course of three daylong training sessions led by an expert in the PRECEDE-PROCEED model, the regional screening program coordinator, and the AGENAS project scientific coordinator.

Day 1 - the LHAs met the PRECEDE-PROCEED model expert, who gave them a theoretical background and subsequently invited them to attempt a local context analysis using the SWOT (Strengths, Weaknesses, Opportunities, and Threats) method to outline the pros and cons of the audit model.

Day 2 - the regional coordinator presented the new planning application based on the PRECEDE-PROCEED model. The participants practiced using the new application for the entire day and gave suggestions on how to improve it (participants were given 1 month after the end of the course to send further suggestions).

Day 3 - a meeting was held to receive suggestions from LHAs. At the end of the training course and after the completion of an assessment test, the participants were credited as ‘auditors.’

In 2017, the DGW-LR officially asked the LHAs to use the application and implemented the first audit cycle. Here are reported the quantitative results of the first year of this audit. The results section reports also a comparison between the planning reports produced by the LHA in 2017 using the PRECEDE-PROCEED application and those produced by the LHA in 2016 when producing a planning report was mandatory but no audit framework was adopted. The comparison is based on a content analysis; DGW-LR analyzed clarity and completeness of each plan: analysis of the weakness of the program, description of the improvement actions, monitoring plan, definition of quantifiable indicators for monitoring implementation and impact. The results were used to develop the audit model described in this paper.

## Results: adapting and testing the model

The project produced two main outputs: an audit system for cancer screening and a new planning application based on the PRECEDE-PROCEED model.

## The audit system for breast cancer screening

The audit cycle starts by setting a date for the site visit via an email to the LHA at least 1 month prior. The audit is led by two external auditors: a regional coordinator and a representative from another LHA.

The visited LHA team has the opportunity to discuss issues, solutions, and outcomes with the two auditors during the visit.

At the end of the site visit, the external auditor writes a report and delivers it within 1 week to the visited LHA. Within 3 weeks from the visit the LHA defines and sends to the DGW-LR an improvement plan. Then the regional coordinator analyzes and evaluates any possible comment and additional actions within 4 weeks from the audit. The regional coordinator monitors the LHA plan at 3–6–9-12 months after the audit. The DGW-LR does not use LHA interim indicators at 3–6-9 months to evaluate LHA performances, only evaluating the results yearly.

## The planning application based on precede-proceed

Starting from the prototypal tool produced by the national project, a software for the user-friendly adoption of the planning application was produced. The application was able to support the audit cycle (the software is attached, a free viewer to use Microsoft Access 2010 is available at https://www.microsoft.com/it-it/download/details.aspx?id=10910).

The original Green & Kreuter The PRECEDE-PROCEED model consists of 9 phases [17]. We applied the phases of the PRECEDE PROCEED model to the setting of organized cancer screening, with some adaptations (Table 2).

**Table 2:** Description of the PRECEDE-PROCEED model according to Green and Kreuter [17] and its tailoring for screening program setting as developed in the region of Lombardy audit model

	original Green & Kreuter	tailored for screening setting
Phase 1 - Social Diagnosis	Identifies and evaluates the social problems which impact the quality of life of a specific population	The General directorate of Welfare of Region of Lombardy identifies outcomes and negotiates targets with Local Health authority (LHA) according to regional targets and local setting
Phase 2 - Epidemiological Diagnosis	Establishes program objective considering the target population	Screening program is described by indicators and epidemiological critical issues are identified
Phase 3 - Behavioral & Environmental Diagnosis	Establishes which factors are linked to health problems and, if modified, can sustain the change process (predisposing, enabling and reinforcing factors).	The LHA can read evidenced-based reviews on which factors are linked to health problems in screening setting and, if modifiable, can sustain the change process
Phase 4 - Education & Organizational Diagnosis	Identifies the presence of the factors categorized in Phase 3	The LHA identifies the presence of evidence-based actions or best practices; analyzes the relationships with key partners; identifies other critical conditions. In this phase organizational critical issues are identified
Phase 5 - Administrative & Policy Diagnosis	Focuses on the administrative and organizational concerns, which must be considered prior to program implementation. Program objective has to be assessed as compatible with the administration and policy	LHA is asked to define priorities (high, medium, low, and very low) and possible solutions for every critical issue. If no solution is feasible, the resources needed must be quantified and sustainability must be verified.
Phase 6 – Implementation	Converts program objectives into actions through policy changes, regulation and organization	Converts program objectives into actions: output and outcome indicators are requested
Phase 7 - Process Evaluation	Evaluates the process of implementation	Evaluates the process of implementation at 3–6–9-12 months from the beginning of the intervention.
Phase 8 - Impact Evaluation	Measures the program effectiveness in terms of intermediate objectives and changes in predisposing, enabling, and reinforcing factors	Measures changes in the main indicators of screening uptake: participation and test coverage
Phase 9 - Outcome Evaluation	Measures change in terms of overall objectives and changes in health, social benefits or the quality of life.	Measures change in terms of efficacy of screening (sensitivity): interval cancer and advanced cancer incidence

### Phase 1: social diagnosis

The objective of the Social Diagnosis is to analyze the setting where the screening will take place, identifying and evaluating the social problems that may influence the achievement of our goal. Since regional authorities have the duty to meet people’s needs through political strategies, we assumed that the background Social Diagnosis had already been performed according to the Italian National Prevention Plan [29].

Furthermore, we considered that the international literature had already defined indicators for the screening setting [30]. Therefore, the Ministry of Health [29] and the DGW-LR identified outcomes considered necessary to evaluate a screening program (i.e., “participation rate”). The DGW-LR and the LHA then negotiated thresholds (Table 3) for all identified outcomes (i.e., participation rate > 60%) to be achieved according to regional objectives and the local setting. The software is flexible and the pre-defined objectives can be changed by adding or removing items from the grid.

**Table 3:** Indicators used for the social (A) and epidemiological diagnosis (B). In many cases there are international, national or locally defined standards. For the indicators included in the social diagnosis specific objectives should be agreed between the General directorate of Welfare of Region of Lombardy (DGW-LR) and the local health authorities (LHA)

Indicators	Italian mandatory standards (LEA)	recommended standards ^a^	standards set by the DGW-LR	Note
% coverage screening	> 55%			A^c^
% advanced cancer screen detected (stage > = 2) at subsequent exams	< 25%			A^c^
%screen detected cancers without staging	< 10%			A^c^
% Participation rate		75%		A^c^
% letters not delivered			< 5%	A
% patients excluded post invitation			< 10%	A
% patients with “waiting for recall <=28 days”		> 90%		A^c^
% people excluded before invitation on total population target^b^			> 0%	A^c^
% people invited on total population target			> 90%	A^c^
% rate of interval cancers 0–11 months			< 10%	B
% rate of interval cancers 12–23 months			< 40%	B
% recall rate first exams		< 7%		A
% recall rate (subsequent examinations		< 5%		A
% screen-detected cancers compared to the total of cancers detected			> 30%	B
average call time (in months)		24	24	A
rate of interval cancers × 1000			< 3	A
sensitivity (proportional incidence)			> 70%	A
sensitivity (screen detected / observed)			> 65%	A
% overall coverage (screening and extra screening)			> 80%	B
% PPV			> 7%	B
% prevalence			to define locally	B
Incidence			to define locally	B
rate of interval cancers ×1000			< 1,5	B
sensitivity (proportional incidence)			> 70%	B
sensitivity (screen detected / observed)			> 99%	B
% coverage 45–49			> 50%	B

A = indicators for social diagnosis; these indicators are mandatory and are defined by the regulatory system. Data for these indicators are collected centrally by the DGW-LR and standards are fixed

B = indicators only for epidemiological diagnosis, data are collected locally by the screening programs

^a^GISMA Italian scientific society of breast screening or European guidelines on Breast cancer

^b^ the majority of screening centres don’t calculate this indicator

^c^ mandatory

### Phase 2: epidemiological diagnosis

The epidemiological diagnosis includes a picture of performance and early outcome indicators of local screening programs and of the burden of disease of breast cancer. The data for this phase are provided by the coordinating center at the regional level and can support a better framing of the intervention objectives and priorities. This phase includes all outcomes considered in Phase 1 and other outcomes useful to describing the burden of disease and the exposure to risk and protective factors in the population (for example breast cancer incidence, spontaneous screening uptake). Starting from the differences between targets to reach (Phase 1) and actual data (Phase 2), the software automatically fills in an EPIDEMIOLOGICAL CRITICAL ISSUES REPORT and a MISSING DATA REPORT. The list of epidemiological items can also be modified.

### Phase 3: behavioral & environmental diagnosis

During the Behavioral & Environmental Diagnosis phase the LHAs are not required to take an active role but they have to review the scientific literature on the uptake processes and the quality of the service provided. The application presents a list of factors that have an impact on the target population concerning cancer screening. Phase 3 is based on the systematic reviews conducted for the Italian HTA report on methods to increase participation in screening programs [13]. The list of factors will be periodically updated as soon as new relevant systematic reviews conducted with appropriate methodology are published; the next update has been planned after the release of the European Recommendations on this topic by the European Commission Initiative on Breast Cancer [31].

### Phase 4: education & organizational diagnosis

The Education & Organizational Diagnosis phase aims to assess the presence of two elements (for each LHA and for each of its hospitals and districts):

- EVIDENCE-BASED (EB) ACTIONS OR BEST PRACTICES: the screening program coordinator of the LHA needs to specify which of the evidence-based actions and best practices have been implemented in the local screening program (for example, invitation with fixed appointment, double-blind reading mammography, counselling). For each EB action or best practice, the LHA has to declare whether EB actions are implemented, their quality level, and any critical issue.
- RELATIONSHIPS WITH KEY PARTNERS: the screening program coordinator of the LHA needs to specify the quality of relationship with stakeholders: the hospital (radiology department, hospital management, pathological anatomy department, etc.), the district (district headquarters, general practitioners), and the community (patient associations, nonprofit organizations, etc.), the departments of LHA (health department, administrative area, etc.). The LHA must declare the quality of its relationship with each stakeholder (highly collaborative, collaborative, not collaborative) and any critical issue.

The users also have the opportunity to add any other conditions that may influence the screening program outcomes which are not in the software form.

The DGW-RL provides a list of evidence-based interventions from systematic reviews [13, 27] and best practices, as well as of possible partners, to focus the LHAs’ attention on some characteristics considered important in managing a screening program, thus performing a stewardship function.

When the LHA certifies that there is no evidence-based intervention (or relationship with key partners) available, or that those available are of low quality, or that there are some issues involved, the software identifies that item as an organizational critical issue. The software automatically composes a list of critical issues. This analysis identifies those conditions that influence any predisposing, enabling, or reinforcing factors. At the end of this phase, the planning application stimulates public health operators to identify which epidemiological indicators are most likely related to one or more organizational issues.

### Phase 5: administrative & policy diagnosis

During the Administrative & Policy Diagnosis phase, the LHA is asked to define priorities (high, medium, low, and very low) and possible solutions for each critical issue. If no solution is feasible, the planning application requires a description and a definition of the resources needed and a verification of the sustainability.

At the end of this phase, the software automatically generates three reports: the ORGANIZATIONAL CRITICAL ISSUES WITH NO SOLUTION report, the UNSUSTAINABLE SOLUTIONS report, and the LOW PRIORITY ISSUES report (Table 4).

**Table 4:** The outputs of the planning application. 7 reports

***REPORT***	***DESCRIPTION***
***1. Epidemiological Critical Issues report***	*summarizes the critical issues identified during the epidemiological analyses, mostly indicators that do not reach the agreed standards (Phase 2).*
***2. Missing Data report***	*highlights the indicators for which the LHA is not able to produce recent data.*
***3. Critical Issues with No Solution report***	*summarizes the identified problems without a solution; to look for a solution the LHA can carry out a literature review and ask the scientific society for help.*
***4. Low Priorities report***	*summarizes the identified problems considered not urgent to solve.*
***5. Unsustainable Solutions report***	*summarizes the problems and related solutions for which the administrative and policy analysis concluded there were no resources available. The screening operator can renegotiate the budget with the LHA general management or can search for external funds (agreements with other agencies, universities, nonprofit organizations,* etc.*).*
***6. Solutions to be Activated report***	*the implementation plan reporting all the medium-high priority problems for which a solution has been identified and resources are available.*
***7. Solutions for Epidemiological Critical Issues report***	*correlates the epidemiological critical issues (identified in report 1) to the solutions to be activated (identified in report 6).*

Local Health Authorities (LHA)

### Phase 6: implementation

During the Implementation phase, the LHA defines the expected outcomes and the process indicators for each solution identified in Phase 5 that was considered medium or high priority and sustainable.

At the end of this phase the software automatically generates two additional reports: the SOLUTIONS TO BE ACTIVATED REPORT and the SOLUTIONS FOR EPIDEMIOLOGICAL CRITICAL ISSUES REPORT (for any epidemiological critical issue found, the identified solutions are described by matching the epidemiological and organizational issues identified in PHASE 4). the LHA can use the reports to draw up a more analytical document with a specific improvement plan.

### Phase 7: process evaluation

In this phase the regional screening program coordinator collects the LHA’s reports on the intervention implementation.

The regional screening coordinator can evaluate the indicators at 3–6–9-12 months from the beginning of the intervention.

### Phases 8–9: impact & outcome evaluation

During the Impact & Outcome Evaluation phase, the LHA evaluates the performance and the early outcome indicators of the screening program (for example, participation rate, detection rate, interval cancer rate, and sensitivity).

All the reports are for internal use, the SOLUTIONS TO BE ACTIVATED REPORT will be used to set up an implementation plan. Furthermore, some of the contents of the reports, particularly the analysis of the relationships with key partners and stakeholders, are strictly confidential. This kind of information can be shared only during the site visit by the regional coordinator; it cannot be made public in official documents.

## Results of the FIRST round of audit adoption

A systematic analysis and synthesis of the action plans produced in 2016 by the LHAs was difficult because the documents presented were extremely heterogeneous in their structure and in presented contents. Nevertheless the analysis of clarity and completeness of these plans showed that the most common weakness was the monitoring plan: completely absent in most cases and not including quantifiable indicators in the remaining. This lack essentially rendered ineffective any attempt by the regional coordinating center and by the DGW-LR to evaluate the plan implementation.

The first round of audit adoption involved 4 out of the 15 screening centres (3 other centres were involved in the pilot phase). The centres were chosen by the DG welfare in relation to the availability of the centres themselves and their performance: 2 centres with excellent performance, 2 centres with poor performance. All centres presented an improvement plan according to the PRECEDE-PROCEED tool framework. Overall, the 4 audits identified 232 critical issues (ex. invitation without fixed appointment; all issues are visible in MS Access attached). After the Administrative & Policy Diagnosis phase, the issues were classified as: 27% low priority issues, 15% not sustainable solutions, 5% issues without a solution. For some of the issues with no sustainable solution, the auditors proposed a sustainable solution during the first feedback that was not found by the visited LHA team; these were added to the final version of the improvement plans (Table 5). The plan included the predefined monitoring form with milestones to be reached at 3, 6, 9, and 12 months, with relative indicators and standards.

**Table 5:** Results of the audits conducted in 2017 in four LHAs using the PRECEDE-PROCEED tool

	EPIDEMIOLOGICAL CRITICAL ISSUES	ORGANIZATIONAL CRITICAL ISSUES	LOW PRIORITY ISSUES	NOT SUSTAINABLE SOLUTIONS	CRITICAL ISSUES WITHOUT A SOLUTION	SOLUTIONS TO BE ACTIVATED	% SOLUTIONS TO BE ACTIVATED / ORGANIZATIONAL CRITICAL ISSUES
LHA A	25	90	14	21	1	54	60%
LHA B	3	62	16	2	3	41	66%
LHA C	4	22	7	2	5	8	36%
LHA D	10	58	25	11	2	20	34%
TOT	42	232	62	36	11	123	53%

## Discussions

### Audit

In this model, planning has a time frame of 1 year in which health professionals can easily understand their roles and define their activity based on annually assigned objectives.

The software and the audit system have been designed as tools for a governance action based on the stewardship principles as defined by the WHO: the “careful and responsible management of the well-being of the population.” [32] The stewardship role of the health systems, which aims at ensuring the promotion of people’s well-being, was adopted by the WHO European region in 2008 with the Tallinn Charter [33] and further elaborated in the Italian context according to the 2010–2013 National Preventive Plan [34]. This role encompasses not only the main stewardship functions at national level but also the function of coordinating the regional and local health authorities.

The first round of audit identified many critical issues: this was expected as it was a systematic analysis of the whole process. It is specified that the criticalities also include potential actions to improve the service. The improvement is agreed with the head of the screening center.

### The PRECEDE-PROCEED tool

The application of the PRECEDE-PROCEED model to screening programs has been validated by several case studies: a systematic review found 8 such studies [35]. All of them were performed in the US and took into account mainly Phase 4 (Education & Organizational Diagnosis), which investigates predisposing, enabling and reinforcing factors. Technical and organizational factors emerged as the most important changeable variables because they act directly on participation (including vulnerable groups) and because they work on highly modifiable factors.

Our adaptation of the PRECEDE-PROCEED model to plan and conduct an intervention aimed at improving screening programs introduced two unique characteristics:

1. given that priorities are mostly given by the European Commission Recommendation [36], by the Italian guidelines and laws [1], and by the national monitoring system defined by the Ministry of Health [29, 30] and by the scientific societies [37, 38] the Social Diagnosis (phase 1) was reduced to the negotiation of thresholds on the main performance and early outcome screening indicators;
2. actions to increase participation in screening programs are already defined by the findings of a recent systematic review sponsored by the Italian Ministry of Health [27].

Regarding the first point, screening programs follow well-defined international guidelines which set several priorities (i.e., target population, standards for recall rate, and sensitivity) that cannot be questioned, at least in the phase of implementing the programs at the local level. The advantage is that these priorities and the guidelines are based on sound studies and strong evidence. It is worth to note, that this approach has been also supported by the new European guidelines, that at least for the 50–69, targeted by the Lombardy screening programs, made a strong recommendation in favor of screening [21].

With regard to the second point, this choice was made in order to reduce the arbitrary selection of possible interventions proposed. It is very important to continually update the items that are proposed in Phase 4.

The plans produced with the tool resulted clearer, more complete and provided with a monitoring including quantifiable indicators. This improvement of the documents produced did not cost more time or workload for screening centers, because the tool automatically framed each single chapter of these documents. The opportunity to speed up the writing of thee improvement plan was particularly appreciated by the screening coordinators already in the 3 days training/tasting workshop.

## Conclusions

With the audit system we want to introduce a “stewardship instrument” to overcome local problems by:

- sharing evidence-based activities to improve screening participation and quality
- standardizing the main characteristics of screening programs in the whole region
- supporting and coordinating the training process of healthcare professionals involved in screening programs

Starting from the application and the audit model developed during this experience, a new audit activity involving all the LHAs was launched in region of Lombardy in 2017. The application helps the LHA screening coordinators to quickly have a clear picture of the situation of the screening program as it highlights both issues and things to do.

The impact of the implementation of the new audit system on screening performance will be evaluated in the next few years.

## Availability and requirements

Project name: Breast Screening PRECEDE PROCEED project.

Project home page: not applicable.

Operating system(s): MS ACCESS (Office 2016 or sup).

Programming language: Microsoft VBA.

Other requirements: Windows 10 or sup.

License:. MS ACCESS (Office 2016 or sup), Windows 10 or sup.

Any restrictions to use by non-academics: it is not intended for use outside cancer screening programs; it is not intended to be used for profit.

## Supplementary Information

**Additional file 1.** Software for precede proceed breast audiT. The software helps health pofessionals to lead an audit in setting of breast screening.

## Abbreviations

GP: General practitioners
AGENAS: National Agency for Regional Health Services
LHA: Italian Local Health Authority
IMH: Italian Minister of Health
DGW-LR: General directorate of Welfare of Region of Lombardy
